# Some Aspects of Intestinal Obstruction

**Published:** 1908-07-25

**Authors:** 


### Surgery.

### SOME ASPECTS OF INTESTINAL OBSTRUCTION.

### THE HISTORY OF A TYPICAL CASE OF CHRONIC OBSTRUCTION (*continued*).

By the time that the paroxysmal attacks of colic and visible coils of peristaltic intestine have become a marked feature, the abdomen is usually distended. The increased proportions of the abdomen depend partly upon an accumulation of intestinal contents, and partly upon meteorism. The degree of distension varies with the site and narrowness of the stenosed area. Meteorism is a condition in which the bowel is distended with the gases of decomposition, and it is more marked if there is much fermentable material in the gut. The gases so formed are unable to pass the stricture, and the damaged vessels of the gut are unable to absorb them. When distension is due to intestinal contents alone the loaded coils of bowel, especially of the colon, tend to gravitate into the loins when the patient is recumbent, and the wide abdomen so produced is not unlike that of ascites.

It has already been mentioned that one of the first symptoms to attract attention in chronic obstruction is the constipation, which gives place at times to a spurious diarrhœa, resulting from a catarrhal enteritis. As a result of the enteritis the fæces passed are often mixed with a considerable quantity of intestinal mucus. Blood and pus are occasionally found in the dejecta and, when present, may be considered as a sign of certain definite varieties of stenosis of the intestine. The passage of blood is most commonly associated with carcinoma and chronic intussusception. It is also found in vascular adenomata and progressive tuberculous ulcers, both of which conditions may give rise to symptoms of obstruction. Hæmorrhoids which are secondary to carcinoma of the rectum account for the blood in a certain number of cases.

Much importance has been attributed by writers to peculiarities in the shape of the solid dejecta passed. Some forms have been likened to articles of common use—*e.g.* pipe-stem or tape-like motions, while others have been compared to sheep's dung and solid stools which show grooves, marks, or spiral indentations on their surface have been described in detail. It may be said at once that they are of little or no value in diagnosis. Treves has pointed out that the shape of the stools is largely, if not entirely, the work of the sphincter ani, and has shown that where this muscle is in an irritable condition, as in fissure, or after an operation for piles, great changes are noticed in the shape of the dejecta. Besides, it is generally assumed that these peculiarities of conformation are given to the stools by their passage through the stenosed area; but as a matter of fact the motion to be passed is finally formed in the ampulla of the rectum and moulded there, so that it is hardly conceivable that a stenosis of the small, or even of the large, intestine could have any permanent influence on the ultimate shape of the dejecta.

In a case in which the symptoms have progressed thus far, the patient exhibits signs of constitutional disturbance. He is enfeebled and wasted, his appetite is impaired, his digestion is upset, he is weakened by the now almost constant paroxysms of colic, and he is being slowly poisoned by the absorption of toxic products of decomposition from his own alimentary canal. If cancer is the primary cause, he shows signs of the condition known as "malignant cachexia." He is losing weight and his skin is inelastic. If pulled up at any part, it returns but slowly to its original position. The urine will be found to contain indoxyl sulphates. The temperature is usually not above normal, but the pulse rate is often increased to as much as 120.

What is the prognosis if enterostenosis is allowed to run its course without interference? It may be said without exaggeration that it is highly unfavourable. The exact degree of gravity varies, of course, in each case with the primary cause. Certain forms of stenosis may undergo a spontaneous cure: this may occur, for example, where the disease is due to the presence of a foreign body, or to a benign neoplasm in the lumen of the alimentary canal; and constriction of the gut by an exudate or by a misplaced organ, may disappear respectively with the absorption of the exudate or the reposition of the organ.

But in the great majority of cases where an operation is not performed, no such favourable result can be hoped for. The duration of the disease will vary in different cases, and a great many factors will determine the length of time that must elapse before death will ensue. The primary cause of the obstruction is an important determining factor. A patient for instance with enterostenosis due to cicatricial stricture will, *cæteris paribus*, live longer than a patient suffering from enterostenosis of carcinomatous origin.

In one class of case the ultimate course of the disease is determined by ulcerative lesions of the intestinal mucous membrane that develop above the stenosis. The ulcers are caused primarily by the mechanical pressure exerted by the stagnant bowel contents, and secondarily by the chemical and bacterial irritants which the fæcal material contains. As a result of these factors, a destruction of the mucous coat is set up, which leads to the form of intestinal ulceration, which is described under the name of the "stercoral" or "decubital" ulcer. Kocher calls them "distension" ulcers (*dehnungs-ge-schwüre*). He believes that the condition is solely due to the disturbance of the intestinal circulation. When this state of affairs has developed, the appearance is very striking. The intestine is usually dilated and sacculated above the stricture, and the mucous membrane in the dilated portion is seen to be covered with a number of these stercoral ulcers. Any one of them may perforate through the bowel wall, and give rise to peritonitis. In this case the prognosis is almost invariably hopeless; for the patient has not only to struggle against one of the most fatal disorders, but he is already weakened to an extreme degree by the pre-existing condition.

